# Identifying sequence features that drive ribosomal association for lncRNA

**DOI:** 10.1186/s12864-018-5275-8

**Published:** 2018-12-31

**Authors:** Chao Zeng, Michiaki Hamada

**Affiliations:** 1 Faculty of Science and Engineering, Waseda University, 55N-06-10, 3-4-1 Okubo Shinjuku-ku, Tokyo, 169-8555 Japan; 2 AIST-Waseda University Computational Bio Big-Data Open Innovation Laboratory (CBBD-OIL), 3-4-1, Okubo Shinjuku-ku, Tokyo, 169-8555 Japan; 3 Artificial Intelligence Research Center, National Institute of Advanced Industrial Science and Technology (AIST), 2-41-6 Aomi, Koto-ku, Tokyo, 135-0064 Japan; 4 Institute for Medical-oriented Structural Biology, Waseda University, 2-2, Wakamatsu-cho Shinjuku-ku, Tokyo, 162-8480 Japan; 5 Graduate School of Medicine, Nippon Medical School, 1-1-5, Sendagi, Bunkyo-ku, Tokyo, 113-8602 Japan

**Keywords:** lncRNA, Ribosome-associated, Sequence feature, Feature selection

## Abstract

**Background:**

With the increasing number of annotated long noncoding RNAs (lncRNAs) from the genome, researchers are continually updating their understanding of lncRNAs. Recently, thousands of lncRNAs have been reported to be associated with ribosomes in mammals. However, their biological functions or mechanisms are still unclear.

**Results:**

In this study, we tried to investigate the sequence features involved in the ribosomal association of lncRNA. We have extracted ninety-nine sequence features corresponding to different biological mechanisms (i.e., RNA splicing, putative ORF, k-mer frequency, RNA modification, RNA secondary structure, and repeat element). An $\mathcal {L}1$-regularized logistic regression model was applied to screen these features. Finally, we obtained fifteen and nine important features for the ribosomal association of human and mouse lncRNAs, respectively.

**Conclusion:**

To our knowledge, this is the first study to characterize ribosome-associated lncRNAs and ribosome-free lncRNAs from the perspective of sequence features. These sequence features that were identified in this study may shed light on the biological mechanism of the ribosomal association and provide important clues for functional analysis of lncRNAs.

**Electronic supplementary material:**

The online version of this article (10.1186/s12864-018-5275-8) contains supplementary material, which is available to authorized users.

## Introduction

With the advancement of high-throughput sequencing technology, the lncRNA population has begun to emerge. In the past few decades, we have had a new understanding of this type of RNA that their number far exceeds the protein-coding gene in human and mouse [1]. However, it is still unclear what function most of the lncRNAs have [2]. Moreover, it is difficult to predict the lncRNA genes from other organisms without sequence characteristics of lncRNAs [1].

Here, we discuss ribosome-associated lncRNAs, which are interacting with the ribosomes although we did not have evidence for their protein translation. Such lncRNAs are considered to have the function of regulating translation [3, 4]. The ribosome-associated lncRNAs are also reported to serve as a source of new peptides [5]. Several individual studies have found encoded peptides from lncRNAs, which have been reviewed in [6]. However, due to the limited number of ribosome-associated lncRNAs, it is difficult to understand in depth what are the essential features (or regulatory elements) included in the lncRNAs that control their association with the ribosome. Characterization of ribosome-associated lncRNAs play a crucial role in understanding the involvement of lncRNA in specific biological functions or which possible regulatory mechanisms.

Ribosome profiling is a technique that collect and read RNA fragments, which are protected by the ribosome. It provides us a way to investigate the genome-wide association of lncRNAs with ribosomes. In the previous work [7], we have analyzed ribosome profiling data and identified 613 ribosome-associated lncRNAs (ribo-lncRNAs) and 746 ribosome-free lncRNAs (noribo-lncRNAs) from human (367 ribo-lncRNAs and 326 noribo-lncRNAs from mouse).

In this study, we investigated which sequence features could distinguish between these two lncRNAs. To our knowledge, this is a first study of characterizing ribosome-associated lncRNAs. Such sequence features identified in this study are possible to be considered as regulatory factors that play an essential role in the ribosomal association.

## Methods

### Datasets and potential features

Ribo-lncRNAs and noribo-lncRNAs were derived from our previous study [7]. We used Blast [8] to remove lncRNAs that share sequences of high similarity. If the sequence similarity between two lncRNAs exceeded 60% (of the shorter one), then it is considered as high similarity and hence the shorter one is discarded. All sequence features considered to affect ribosome association were listed in Additional file 1: Table S1. For each feature column, we imputed missing data by using mean value.

#### Primary/first/upstream ORF

**Fig. 1:**
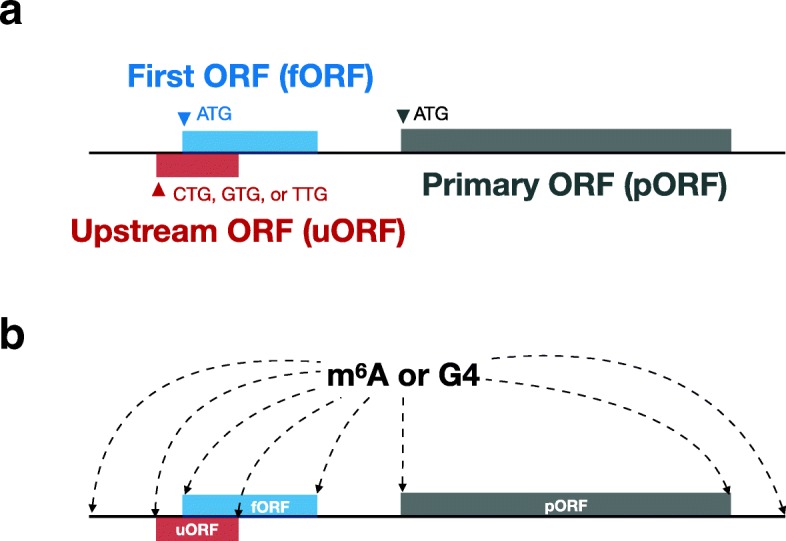
Example of feature extraction. **a** Representation of primary ORF (pORF, gray), first ORF (fORF, blue), and upstream ORF (uORF, red) in a lncRNA. Horizontal line indicates a mature lncRNA, boxes represent putative open reading frames (ORFs) defined on this lncRNA. **b** Relationship (distance) between m^6^A/G4 and transcript initiation site (TIS), transcript termination site (TTS), and starts or ends of u/f/pORF were used as features. Direct distance (bases in log scale) and relative distance (percentage of the length of lncRNA) were considered to express the relationship

We defined three different types of putative open reading frames (ORFs) on a lncRNA (Fig. 1). A primary ORF (pORF) is the longest ORF starting with ATG. A first ORF (fORF) starts with ATG and is closest to the 5 ^′^ end of the lncRNA. An upstream ORF (uORF) starts with a near-cognate initiation site (i.e. CTG, GTG, or TTG [9]). Here, the uORF is considered only when an existing pORF located in the lncRNA; the beginning and end of uORF should be upstream of the pORF. These three types of ORFs above are all terminated with a TAG, TGA, or TAA. In addition, the upstream ORF overlapping with the primary ORF was not analyzed in this study.

#### Context/trimer/hexamer score

For the three types of ORFs mentioned above, we defined three scores based on frequency ratio between ribo-lncRNAs and noribo-lncRNAs. Context sequence score of ORF start (hereinafter abbreviated as “context score”) is the sum of frequency ratios of nucleotides at -6 to +3 positions relative to the ORF start. Trimer score and hexamer score are summed frequency ratios of trinucleotide or hexanucleotide, respectively, during ORFs. These three metrics can be calculated using the following formula (which is also applied to assess coding potential in CPAT[10]):

1 $$\begin{array}{@{}rcl@{}} \mathrm{Context/Trimer/Hexamer\ score} \!= \! \frac{1}{n}\sum\limits_{i=1}^{n}\log\left(\frac{F\left(x_{i}\right)}{{F}'\left(x_{i}\right)}\right) \end{array} $$

where, for context score, *x*_*i*_∈[*A,C*,*G,T*] represents the nucleotide at the i-th position while *i* indicates the index of the relative position above (*i* = 1.. 10). *F*(·) and *F*^′^(·) are the occurrence frequencies of position-specific nucleotide in categories of ribo-lncRNA and noribo-lncRNA, respectively. For trimer score and hexamer score, ORF sequence is converted into a sequence of length *n* in units of trinucleotide and hexanucleotide, respectively. Thus, *x*_*i*_ represents the unit (trimer or hexamer), *F*(·) and *F*^′^(·) are the occurrence frequencies of unit in ribo-lncRNAs and noribo-lncRNAs, respectively. Both *F*(·) and *F*^′^(·) need to be calculated in advance from a control dataset to generate a lookup table. Hence, we randomly selected 5000 CDS sequences to calculate *F*(·) and shuffled those sequences to generated *F*^′^(·).

#### Stem probability

A higher stem probability means a stronger RNA secondary structure in this context. To investigate whether RNA secondary structure affects the ribosomal association, we used ParasoR [11], which is specifically designed for RNA secondary structure prediction of numerous and long RNAs, to predict the stem probability of each base in a lncRNA. We set the parameter −*constraint* to *N*−1, where *N* is the length of the lncRNA, in order to consider all possible base pairs during the lncRNA. Except it was an extreme long (>9500nt) RNA, we used the default parameter (−*constraint*=200) to guarantee the prediction result in a limited time.

#### N^6^-Methyladenosine modification, G-quadruplex, and repeat element

We used SRAMP [12] to predict N^6^-Methyladenosine modification (m^6^A) sites in a lncRNA. G-quadruplex (G4) segments were predicted by using QGRS [13]. G4 element with G-score ≥ 30 is considered as a stable G-quadruplex structure. Transposon elements (TEs) annotations were obtained from RepeatMasker [14]. We used the repeat library (build on 20140131) that mapped to human (hg19) and mouse (mm10), respectively. Repeat elements annotated as simple repeats, low-complexity, or non-coding RNA were removed.

### $\mathcal {L}1$-regularized logistic regression

Logistic regression (LR) model [15] can be used as a binary classifier which applies a logistic function to turn linear predictions to [0, 1]. Given a set of labeled training data *X* (feature vectors) and their labels *y* (i.e. 0 and 1 indicates noribo-lncRNA and ribo-lncRNA, respectively), LR model seeks to minimize the loss (or objective) function:

2 $$\begin{array}{@{}rcl@{}} \min_{w,c}\left \| w \right \|_{1} + C\sum\limits_{i=1}^{n}\log\left(\exp\left(-y_{i}\left(X_{i}^{T}w + c\right)\right) + 1\right). \end{array} $$

To avoid the over-fitting, in which a complicate (many parameters and parameters with a large variance) model can perform perfectly on training dataset but badly on testing dataset, a regularization term (∥*w*∥_1_) was used to control the complexity (i.e. the number and the values of parameters) of model. Moreover, $\mathcal {L}1$-based regularization drives parameters to zero, which is a natural process of feature selection. After training the LR model, we get a small number of features with non-zero coefficients. Since the feature value has been scaled in the same range, the absolute value of the coefficient represents how much the change of this feature has an effect on the prediction of the model, and can be used to express the importance of this feature in classification. The choice of using the model is based on following reasons: First, the model uses a logistic function to transform the prediction results to a range of 0 to 1, which is suitable for a two-class problem involved in this study; Second, $\mathcal {L}1$-regularization drives the model to tend to adopt a sparse feature space during training, that is, the coefficients of many features will be zero, resulting in the model naturally selects features for us; Finally, a linear combination of all features is considered in the model. Thus, a positive/negative sign of the coefficient of the feature indicates that a positive/negative correlation with the result of prediction (i.e. ribo-lncRNA), and an absolute value of the coefficient can be used to describe the importance of the responding feature.

Feature selection by using the $\mathcal {L}1$-regularized logistic model becomes a univariate problem of how to select a hyperparameter *C*. Here, *C* represents the inverse of regularization strength. As *C* is increased, the number of features with non-zero coefficients is increased, and the model becomes more complicated. Thus, the criteria used in this study is that the most appropriate *C* should be to select fewer non-zero feature coefficients while still ensuring that the model has relatively high prediction accuracy. For this purpose, we divided all data into a training set and test set in a ratio of 80:20, and the training set was further applied for 5 fold cross validation. When we determine a value of *C*, the model optimizes all the feature coefficients on the training set. Then the performance of the optimized model was evaluated on the test set using accuracy metric:

3 $$ \text{Accuracy} = \frac{TP + TN}{TP + TN + FP + FN}  $$

where, *TP* is number of true positives, *FP* is number of false positives, *TN* is number of true negatives, and *FP* is number of false negatives. We used the Python scikit-learn library [16] to perform all the machine learning processes mentioned above.

## Results

### Defining ninety-nine features from lncRNA sequence

We considered factors that may cause lncRNA to associate with ribosome in terms of RNA splicing, putative ORF, k-mer frequency, RNA secondary structure, RNA modification, and repeat elements. A full list of extracted features is included in Additional file 1: Table S1.

#### RNA splicing

To investigate the relationship between splicing and ribosomal association, we mainly examined length and G + C content of intron and exon. Because the first exon and intron was important for alternative splicing [17–19], their length and G + C content were also included in our feature set.

#### Putative ORF and k-mer frequency

We first defined three types of ORFs (primary, first, and upstream), then extracted sequence features based on them (see “Methods” section for more details). As shown in Fig. 1a, pORF is the longest ORF which is considered most frequently as a possible translated region; fORF is the ORF closest to the 5 ^′^ end of the lncRNA which was selected because of the first-ATG rule [20]; uORF locates in the upstream of the primary ORF starting with near-cognate initiation site (i.e., CTG, GTG, or TTG). Other ORFs located inside or in the downstream of the primary ORF were excluded to ensure the simplicity of the problem.

ORF length is a discriminating feature for coding and non-coding RNAs[10], hence we questioned whether this feature can also contribute to the detection of ribosome-associated lncRNA. As it was reported that 3 ^′^ UTR length may regulate the translation efficiency [21] and 5 ^′^ UTR may contain RNA modification [22] or regulatory motif (e.g., G-quadruplex [23]), they were also considered in this investigation. Moreover, we used trimer score and hexamer score to assess whether the codon usage and bi-codon frequency were similar to CDS. To calculate trimer (or hexamer) score, we first randomly selected 5000 CDSs as active ORF reference and randomly shuffled their sequences as inactive ORF reference (Additional files 1 and 2). Each trimer (or hexamer) has a weight, which is the ratio of its occurrence frequency in the two reference groups. For a given putative ORF, we calculated the weight of all trimers (or hexamers), and then took the mean to represent its trimer (or hexamer) score (see “Methods” section). Thus, trimer (or hexamer) score measures the degree of trimer (or hexamer) usage bias in a specified putative ORF. A positive score indicates a possible active ORF, whereas a negative score indicates an inactive one.

A consensus sequence, termed Kozak sequence, surrounds the start codon in eukaryotic mRNAs and is reported to promote the translation initiation [24]. To take this into account, we developed context score to compare sequence motif surrounding the putative ORF start with that surrounding the start codons from mRNAs. The calculation of context score is similar to that of the trimer/hexamer score above. We calculated the weight of each base at -6 to +1 positions relative to the start codon. Indeed, we observed the Kozak sequence motif in this position-specific weight matrix (Additional file 1: Figure S1). Hence, the higher the context score, the more similar to the Kozak sequence.

#### RNA secondary structure

We considered the RNA stem probability as a metric of RNA secondary structure, and then defined RNA structure features with respect to 5 ^′^/3 ^′^ UTRs and ORF. Both experimental and computational studies have observed that ORF sequences were more structured comparing with other regions in the mRNAs [11, 25], and a change of RNA secondary structure can be often observed surrounding the start and the stop codon. Thus, we calculated the RNA stem probability which indicates the likelihood that each base is included in a RNA stem structure across the full RNA sequence. Then we could extract averaged stem probabilities for distinct regions corresponding to pre-defined putative ORFs. Furthermore, we proposed that a stem probability ratio of 5 ^′^ UTR to ORF is needed to quantify the RNA structure changes between these two regions. Similarly, we also defined the ratio between 3 ^′^ UTR and ORF.

G4 is a four-stranded helical structure which can form in RNA and may be involve in translational control. Although the study of G4 is still in its infancy, it is inferred from its stable RNA secondary structure that G4 may block the translational regulation of the relevant site when it is close to the 5 ^′^ cap structure, the start codon, and the stop codon [26]. Additionally, G4 may also provide a cap-independent initial entry for translation initiation factors, thereby facilitating RNA translation [23, 26]. To explore whether G4 affects the association of lncRNAs with the ribosome, we first predicted the possible G4 structure in lncRNAs using QGRS [13], and then considered the relative positions of these G4s relative to transcription initiation site (TIS), transcription termination site (TTS), and the start and end of the putative ORF (Fig. 1b). In addition, for the definition of relative position, we used two kinds of measurement methods: direct distance and relative distance. Direct distance represents the number of nucleotides on the RNA between the G4 and the target site mentioned above. Relative distance is a measure of the direct distance normalized to the total length of the RNA, to prevent possible bias of different RNA lengths.

#### RNA modification and repeat element

We utilized SRAMP [12] to predict where an m^6^A might occur in a lncRNA, and calculated the direct and relative distances of the m^6^A to various locations (i.e. TIS, TTS, and start/stop codons) as features. This is because previous studies have found that the m^6^A is often enriched in a 5 ^′^ UTR or in a 3 ^′^ UTR neighboring stop codon [27, 28]. The m^6^A that located in the 5 ^′^ UTR can promote cap-independent translation [22], while the m^6^A located around the stop codon may promote translation initiation by a binding protein. Finally, we were interested in whether the lncRNA contains a particular repeat element as a binarized feature. For example, Alu element is reported to be related to the cellular localization of lncRNAs [29], and our previous work have shown that the ribosomal association of lncRNAs,indeed, is positively correlated with the nuclear localization of lncRNAs. SINEB2, which is one of SINE (short interspersed nuclear element) repeat sequence, is reported to be associated with the up-regulated translation [30]. Hence, we do not rule out that SINE or other repeat elements may have the potential to regulate the ribosomal association of lncRNA.

**Fig. 2:**
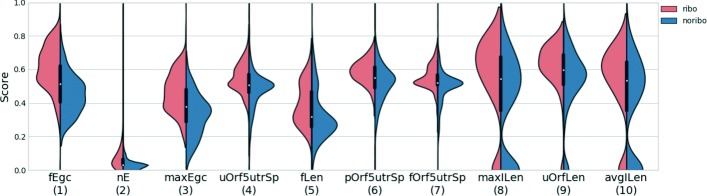
Distribution of top 10 feature scores in human. Each feature was ranked by -log(KS *p*-value), in which KS represents two samples Kolmogorov-Smirnov test between ribo-lncRNAs (red) and noribo-lncRNAs (blue)

Figure 2 shows the distribution of some features in ribo-lncRNA and noribo-lncRNA in human (see Additional file 1: Figures S2–S3 for the distribution of all features; the meaning of the features are described in Table 1). According to the KS importance (described below) of each feature, we ranked all the features from high to low in the figure. Interestingly, if only one feature was chosen to distinguish the two types of lncRNAs, the GC content of the first exon (fEgc) was the most discriminating feature. We observed that ribo-lncRNAs tend to have a higher GC content in their first exons both in human and mouse. Here, all feature values were transformed in a range of 0 to 1. Then, we used two-sample Kolmogorov—Smirnov (KS) statistic [31] to examine the ability of each feature to separate the two types of lncRNAs (KS importance). The two-sample KS statistic is a non-parametric test to compare two groups of samples. When a feature has a significant difference between the two groups of lncRNAs, a smaller *P* value will be obtained in the two-sample KS statistic. If we only consider the effect of an individual feature, we can rank the features according to the statistical significance level (-log *P* value) from high to low. This method can be used for feature selection. Since it only independently assesses the importance of a single feature, it is also referred to as a filter method. This method is fast and straightforward and works well in many scenarios, but it cannot consider the combination of various features in the classification. For this purpose, we will carry out a more systematic screening of these extracted features as below.

**Table 1:** Statistics of dataset used in this study

	Human		Mouse	
	Original	Reduced	Original	Reduced
ribo-lncRNA	613	**487**	367	**279**
noribo-lncRNA	746	**681**	326	**300**
Total	1359	**1168**	693	**579**

The “reduced” column shows the number of lncRNAs after removing sequences of high similarity

### Removing high redundant features

**Fig. 3:**
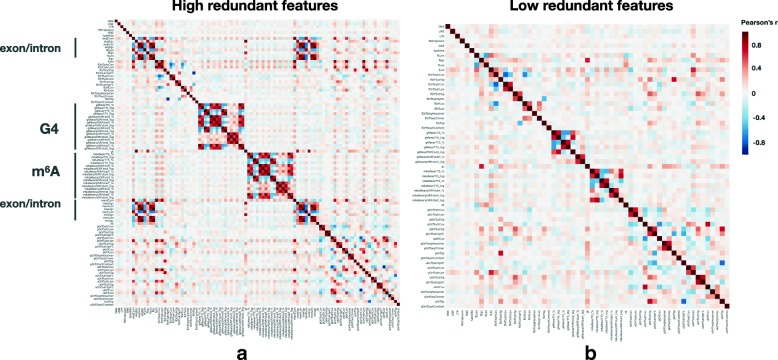
Correlations (r) of features indicate redundant features in human. **a** Correlations of all extracted features show that features of several sub-regions are highly correlated (redundant). **b** After removing high redundant (|*r*|>0.8) features, we obtained a low redundant feature set for further analysis in this study

One feature is considered to be redundant in the presence of another related feature with which is strongly correlated and can be removed without incurring much loss of information. To eliminate redundant features, we investigated the correlation coefficient between all features (Fig. 3a). The results show that the high redundant (|*r*|>0.8) features are mainly clustered on exon/intron, G4, and m^6^A in the form of length or distance. For example, in human, there is a high correlation between the lengths of a transcript and the longest exon in the transcript; the lengths of a pORF and the downstream 5 ^′^ UTR, and the length of a 3 ^′^ UTR of fORF and that of an uORF (*r*>0.8, Additional file 2). The distance of m^6^A relative to the transcript 5 ^′^ end was highly correlated with its distance to the start of uORF (*r*=0.949). Similarly, there is a high correlation between the distance of G4 relative to the start of fORF and its distance to the start of uORF (*r*=0.928). We also observed similar results in mouse (Additional file 1: Figure S4a and Additional file 3).

After removing redundant features, we prepared low redundant features which were ready for a further feature selection. We removed one feature from each pair of redundant features to obtain the low redundant features (Additional files 1 and 2). Then, 59 and 55 sequence features were remained in the human and mouse, respectively. A list of low redundant features is given in Additional file 1: Table S2. Figure 3b shows the correlation coefficient matrix between human low redundant features (see Additional file 1: Figure S4b for mouse). Although there are still some weak correlations between some features (e.g., the direct distance and the relative distance between m^6^A and TIS), filtering of highly correlated features allows us to consider the importance of each feature more distinctly.

### Feature selection by $\mathcal {L}1$-regularized logistic regression

**Fig. 4:**
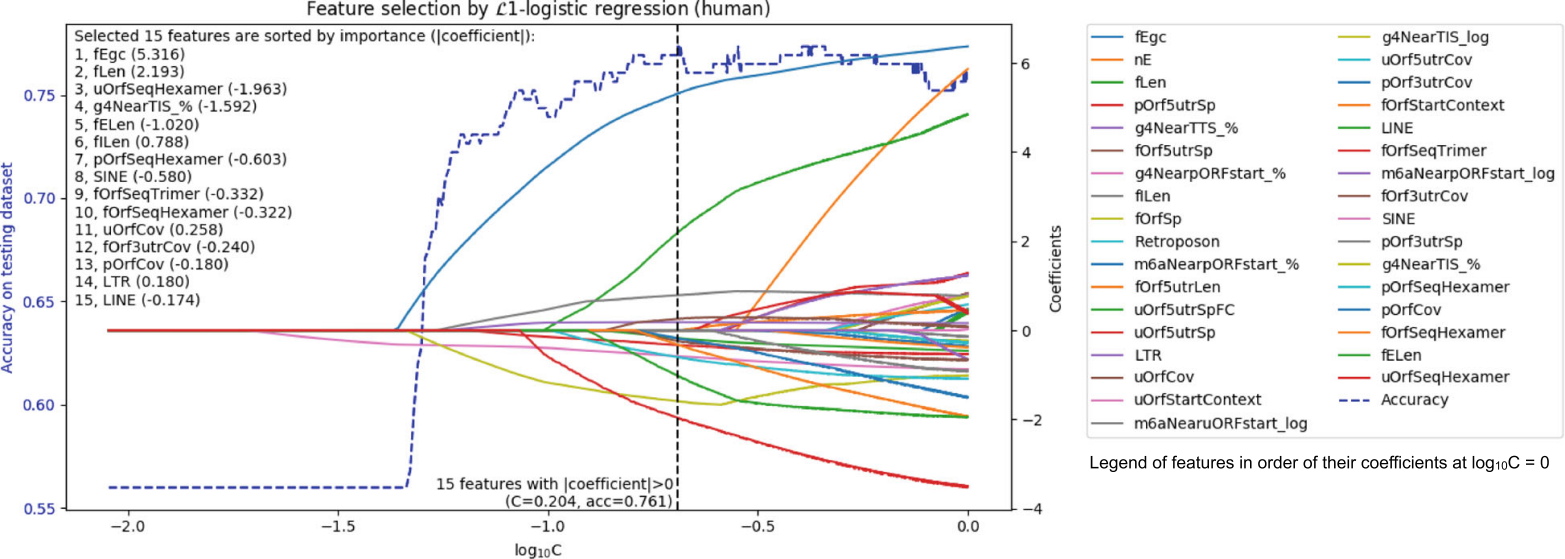
Feature selection by using $\mathcal {L}1$-logistic regression in human. Total data was randomly separated into 80% for training the model and 20% for the calculation of accuracy (blue dashed line, left y-axis). On the x-axis, *C* indicates the inverse of regularization strength. As *C* is increased, the number of features with non-zero coefficients (right y-axis) is increased and the model becomes more complicated. The black dashed line shows the final model chosen in this study, and outputs 15 features with non-zero coefficients. These features were ranked by the absolute value of coefficient, which represents the importance for prediction, and shown in the upper left

Feature selection by using the $\mathcal {L}1$-regularized logistic model becomes a problem of how to select a hyperparameter *C* (see “Methods” section). As shown in Fig. 4, in a range of [0.01,1], we increased the value of *C* in steps of 0.001 and finally obtained the function between the *C* and the feature coefficients (colored solid lines), and the accuracy of prediction (blue dashed line). When the value of *C* is very small, the regularization strength is enormous and all of the feature coefficients are zeros, which means that no feature will be used as a predictor. At this time, the prediction accuracy implies that we predict all the results as positives (i.e., ribo-lncRNAs), which exactly reflects the proportion of positives in the test dataset. In human, for instance, the accuracy at this time is about 55%, which means that the number of positives and negatives in our test dataset is well-balanced. As the value of *C* increases, the more coefficients of the features turn to be non-zero, the prediction accuracy from the beginning of the rapid growth, to later stability or even a decrease. According to the criteria mentioned above, we choose *C*=0.257 at the black vertical line in Fig. 4, and the prediction accuracy at this time is 0.828. The features with non-zero coefficients corresponding to this are the critical features that we finally screen out. We can see that even if we continue to increase the value of *C* (to apply more features), this prediction accuracy has not improved considerably.

Taken together, we identified fifteen crucial sequence features of ribosomal association for human lncRNAs (nine for mouse lncRNAs). A list sorted by the importance of the crucial features is shown in the upper left corner of Fig. 4 (see Additional file 1: Figure S5 for mouse).

## Discussions

By comparing the sequence features of the ribosomal association that we have identified in human and mouse lncRNAs, it is observed that seven features are conserved between the two species. It means that these common features may involve in the biological mechanisms of ribosomal association. Meanwhile, eight (human) and two (mouse) species-specific features are observed, which may involve species-specific regulatory mechanisms of the ribosomal association. In the following subsections, we discuss these features from the aspects of conserved and species-specific.

### Conserved features

Conserved features include the fEgc, fELen, fILen, fOrfSeqHexamer, fOrf3utrCov, uOrfSeqHexamer, and LTR. Out of them, fEgc, fILen and LTR were positively correlated with the ribosomal association, while others vice versa. We observed that the G + C content and the length of the first exon had a high positive and negative correlation with the ribosomal association of lncRNA respectively. This finding matches with the results a study regarding the correlation between ribosome-associated mRNA and CDS [32]. High G + C content may indicate the occurrence of unexpected selection on ribosome-associated lncRNAs [33].

We could also observe that the longer the first intron, the more favorable lncRNAs are associated with the ribosome. The selection forces of intron-dependent nonsense-mediated RNA decay (NMD) on the first intron may be a reason for this situation [34]. This phenomenon is common among protein-coding genes, and a simple hypothesis is that longer introns are more likely to contain certain motifs [18], and these motifs may have essential factors that promote ribosomal association.

Surprisingly, the hexamer frequencies, which were used to assess the coding potential, of the first ORF and the first non-ATG ORF were inversely related to the ribosomal association. The reasons for this can be considered from two aspects: First, even if the ribosome has translation event on these two ORFs, the probability of detection of this event is low due to the length of the two ORFs is relatively shorter than that of the primary ORF. Moreover, the stronger the translation activity on these two ORFs will directly affect the ribosomal initiation of downstream pORFs, resulting in the failure of ribosome association on pORF to be detected. Second, we argue that the ribosomal association mentioned here not be the same as the ribosomal translation. The ribosome may use regulatory mechanisms other than the properties of the CDS sequence, to associate with particular RNAs (e.g., internal ribosomal entry site). Note that we did remove lncRNAs with translation potential when collecting ribosome-associated lncRNAs.

The results of human and mouse consistently demonstrated that lncRNAs containing a long terminal repeat (LTR), are more likely to associate with the ribosome. LTR is often used as a tool when viruses insert genetic material into a host genome. A well-known example of LTR is the human immunodeficiency virus (HIV), in which the LTR contains promoter, enhancer and other functional sequence elements [35]. Furthermore, our results indicate that LTR may be a functional element that promotes the ribosomal association or even translation.

### Species-specific features

In human, the lncRNA length and the length of the non-ATG ORF are positively correlated with the ribosomal association. The remaining six features — the length and the hexamer frequency of the pORF, the trimer frequency of the fORF, the distance between G4 and TIS, and whether it contains LINE or SINE — have a negative correlation with the ribosomal association. In mouse, there are only two species-specific features — the RNA secondary structure of 3 ^′^ UTR of pORF and the distance between m^6^A and transcript 3 ^′^ end — have a negative correlation with the ribosomal association.

Transcript length is one among the important features while distinguishing between protein-coding RNA and noncoding RNA [10]. As expected, this feature can also be used to distinguish ribo-lncRNA and noribo-lncRNA to some extent. The longer the transcript, the higher the probability that it may be associated with the ribosome (according to statistical point of view). Besides, the longer the sequence, the more likely it is to include functional motifs that promote ribosomal association. On the ORF, the features of the trimer/hexamer frequency and the length may be similar to those discussed above.

In contrast to LTR, SINE and LINE (long interspersed nuclear element) are more likely to appear in a ribosomefree lncRNA. This result is consistent with a report that Alu (a type of SINE) can drive the lncRNA in the nucleus [29]. We argue whether there is a set of complementary mechanisms controlling lncRNAs in the cytoplasm and nucleus by applying LTR and SINE/LINE. A systematic analysis of how these repeat elements affect the localization of lncRNAs can help us to understand the role of repeat elements in the evolution of genome, and the biological functions and mechanisms that lncRNAs may have involved.

G4 affects the ribosomal association when approaching transcript 5 ^′^ end. This result is also discussed in many studies [23, 26]. Meanwhile, it further exhibits that the biological regulation of RNA in the secondary structure level. We observed that m^6^A modification appears around transcript 3 ^′^ end affecting the ribosomal association. Wang and colleagues mentioned that m^6^A might form an RNA loop near the stop codon that brings the distance between the start and the stop codons closer to promote the translation efficiency [36]. However, the m^6^A near TTS may hinder the formation of this mechanism. Finally, we compared mRNA with ribo-lncRNA and noribo-lncRNA (Additional file 1: Figures S6–S7). It can be observed that in human, the length of the transcript can indeed be used to distinguish between lncRNA and mRNA. Additionally, we noticed that 5 ^′^/3 ^′^ UTR of ribo-lncRNA seems to have a stronger RNA secondary structure compared with that of mRNA. In mouse, noribo-lncRNA has less number of exons compared with mRNA, which means the corresponding gene model is more straightforward.

## Conclusion

This study analyzed the features of the ribosome-associated lncRNA at the level of sequence. Using the ribo-lncRNAs (ribosome-associated lncRNAs) and noribo-lncRNAs (ribosome-free lncRNAs) collected from human and mouse in our previous study [7], we analyzed which features are most important for distinguishing between the ribo-lncRNAs and the noribo-lncRNAs. Considering the reasons that a lncRNA may be involved in the ribosomal association, we mainly define sequence features based on distinct dimensions from several aspects such as RNA splicing, putative ORF, k-mer frequency, RNA secondary structure, RNA modification, and repeat element. Highly redundant features are removed by analyzing the correlation coefficient of each pair of features. Then, based on the $\mathcal {L}1$-regularized logistic regression model, we performed a feature selection while training feature parameters. Finally, we obtained fifteen and nine essential features for distinguishing between ribo-lncRNA and noribo-lncRNA from human and mouse, respectively, and discussed possible relationships between these features and the ribosomal association. To the best of our knowledge, this should be the first study of how to further divide ribo-lncRNA and noribo-lncRNA from the perspective of sequence features. This research describes how to extract sequence features to study lncRNAs and other biological phenotypes (e.g., subcellular localization), which provide research ideas for similar work. Moreover, the analysis of these sequence features has a critical reference value for us to understand further the ribosomal association, which is still an unknown mechanism, for lncRNA.

## Additional files

Additional file 1Supplementary materials (figures and tables). Figure S1 Context scoring matrix measures the similarity of Kozak sequence (human). Figure S2 Distribution of all feature scores in human. Figure S3 Distribution of all feature scores in mouse. Figure S4 Correlations (r) of features indicates redundant features in mouse. Figure S5 Feature selection by using $\mathcal {L}1$-logistic regression in mouse. Figure S6 Training $\mathcal {L}1$-logistic regression model on the dataset of **a** ribo-lncRNAs and mRNAs; **b** noribo-lncRNAs and mRNAs in human. Figure S7 Training $\mathcal {L}1$-logistic regression model on the dataset of **a** ribo-lncRNAs and mRNAs; **b** noribo-lncRNAs and mRNAs in mouse. Table S1 Sequence features were considered to influence the ribosomal association. Table S2 Low-redundant features in human and mouse. (PDF 3697 kb)

Additional file 2 Raw data for human. (ZIP 9950 kb)

Additional file 3 Raw data for mouse. (ZIP 5080 kb)

## Abbreviations

CDS: Coding sequence
FN: Number of false negatives
fORF: Putative first ORF in lncRNA
FP: Number of false positives
G4: G-quadruplex
HIV: Human immunodeficiency virus
LINE: Long interspersed nuclear element
lncRNA: Long noncoding RNA
LTR: Long terminal repeat
m^6^A: N6-Methyladenosine modification
NMD: Nonsense-mediated RNA decay
noribo-lncRNA: Ribosome-free lncRNA
ORF: Open reading frame
pORF: Putative primary ORF in lncRNA
ribo-lncRNA: Ribosome-associated lncRNA
SINE: Short interspersed nuclear element
TE: Transposon element
TIS: Transcript initiation site
TN: Number of true negatives
TP: Number of true positives
TTS: Transcript termination site
uORF: Putative upstream ORF in lncRNA
UTR: Untranslated region
