# Supplementary material for: Identifying sequence features that drive ribosomal association for lncRNA
Source: BMC Genomics. 2018 Dec 31;19(Suppl 10):906. doi: 10.1186/s12864-018-5275-8 (PMC6311901; doi:10.1186/s12864-018-5275-8)
Supplement: Supplementary file 1 — Supplementary materials (figures and tables). Figure S1 Context scoring matrix measures the similarity of Kozak sequence (human). Figure S2 Distribution of all feature scores in human. Figure S3 Distribution of all feature scores in mouse. Figure S4 Correlations (r) of features indicates redundant features in mouse. Figure S5 Feature selection by using $\mathcal {L}1$ℒ1-logistic regression in mouse. Figure S6 Training $\mathcal {L}1$ℒ1-logistic regression model on the dataset of a ribo-lncRNAs and mRNAs; b noribo-lncRNAs and mRNAs in human. Figure S7 Training $\mathcal {L}1$ℒ1-logistic regression model on the dataset of a ribo-lncRNAs and mRNAs; b noribo-lncRNAs and mRNAs in mouse. Table S1 Sequence features were considered to influence the ribosomal association. Table S2 Low-redundant features in human and mouse. (PDF 3697 kb) [file 12864_2018_5275_MOESM1_ESM.pdf]

Supplementary materials

- **Figure S1** Context scoring matrix measures the similarity of Kozak sequence (human)
- **Figure S2** Distribution of all feature scores in human
- **Figure S3** Distribution of all feature scores in mouse
- **Figure S4** Correlations (r) of features indicates redundant features in mouse
- **Figure S5** Feature selection by using $\mathcal{L}$1-logistic regression in mouse
- **Figure S6** Training $\mathcal{L}$1-logistic regression model on the dataset of **a** ribo-lncRNAs and mRNAs; **b** noribo-lncRNAs and mRNAs in human
- **Figure S7** Training $\mathcal{L}$1-logistic regression model on the dataset of **a** ribo-lncRNAs and mRNAs; **b** noribo-lncRNAs and mRNAs in mouse
- **Table S1.** Sequence features were considered to influence the ribosomal association
- **Table S2.** Low-redundant features in human and mouse

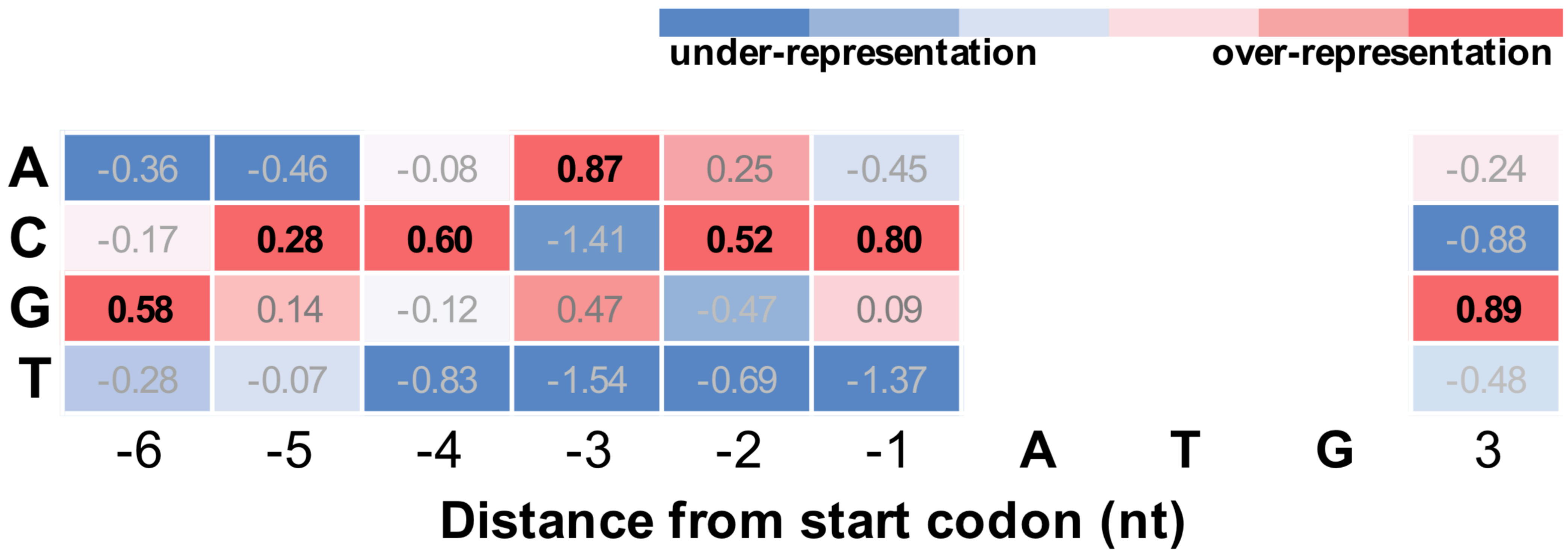

Figure S1 Context scoring matrix measures the similarity of Kozak sequence (human). We calculated the context scoring matrix from 5,000 CDSs (see "Method"). This indicates a Kozak sequence motif (gcc[a/g]ccATGg) surrounding the start codon.

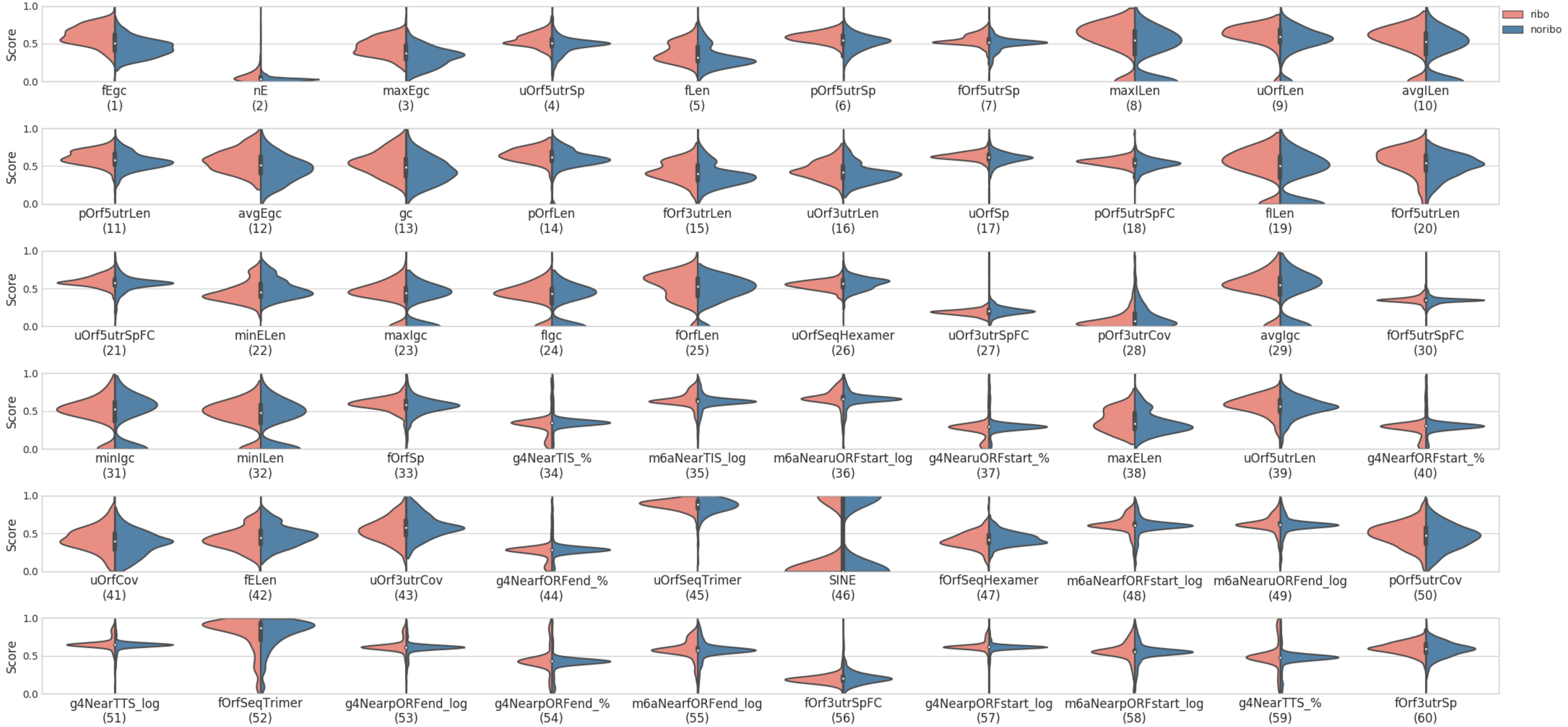

Figure S2 Distribution of all feature scores in human. Each feature was ranked by -log(KS p-value), in which KS represents two samples Kolmogorov-Smirnov test between ribo-lncRNAs (red) and noribo-lncRNAs (blue).

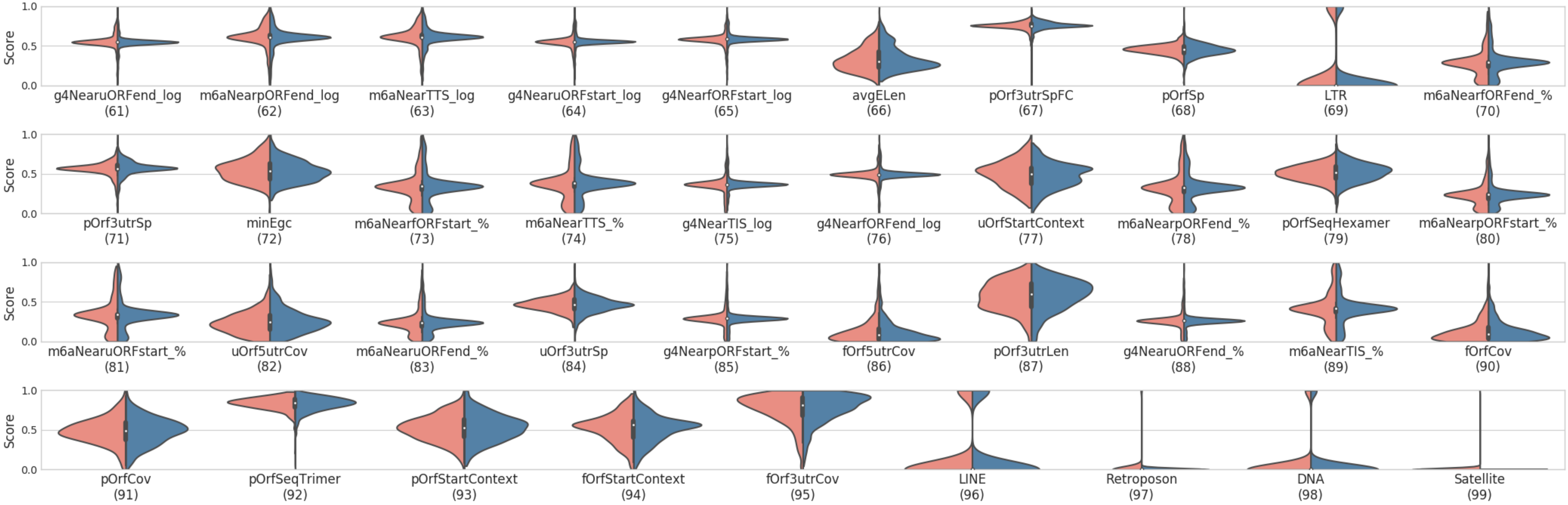

Figure S2 Distribution of all feature scores in human (continued).

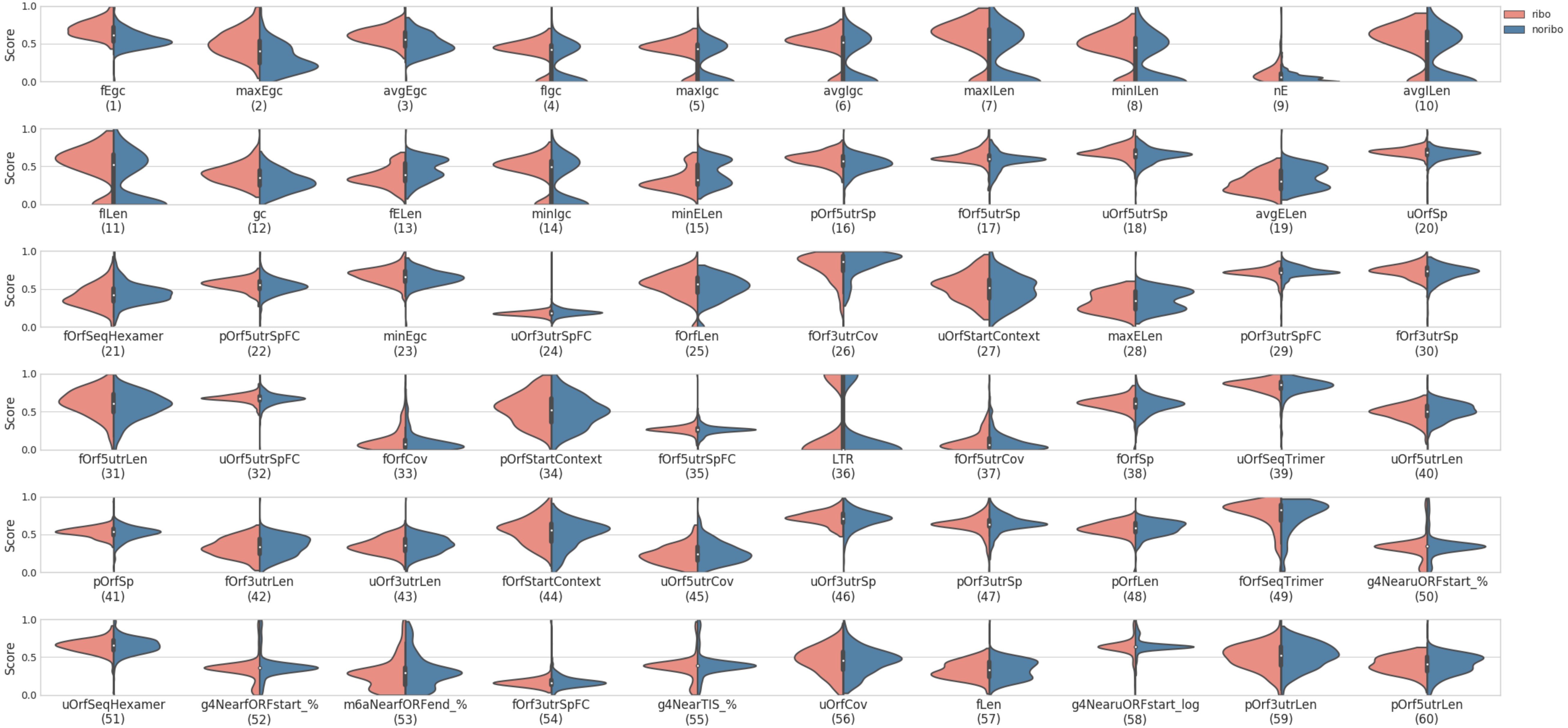

Figure S3 Distribution of all feature scores in mouse. Each feature was ranked by -log(KS p-value), in which KS represents two samples Kolmogorov-Smirnov test between ribo-lncRNAs (red) and noribo-lncRNAs (blue).

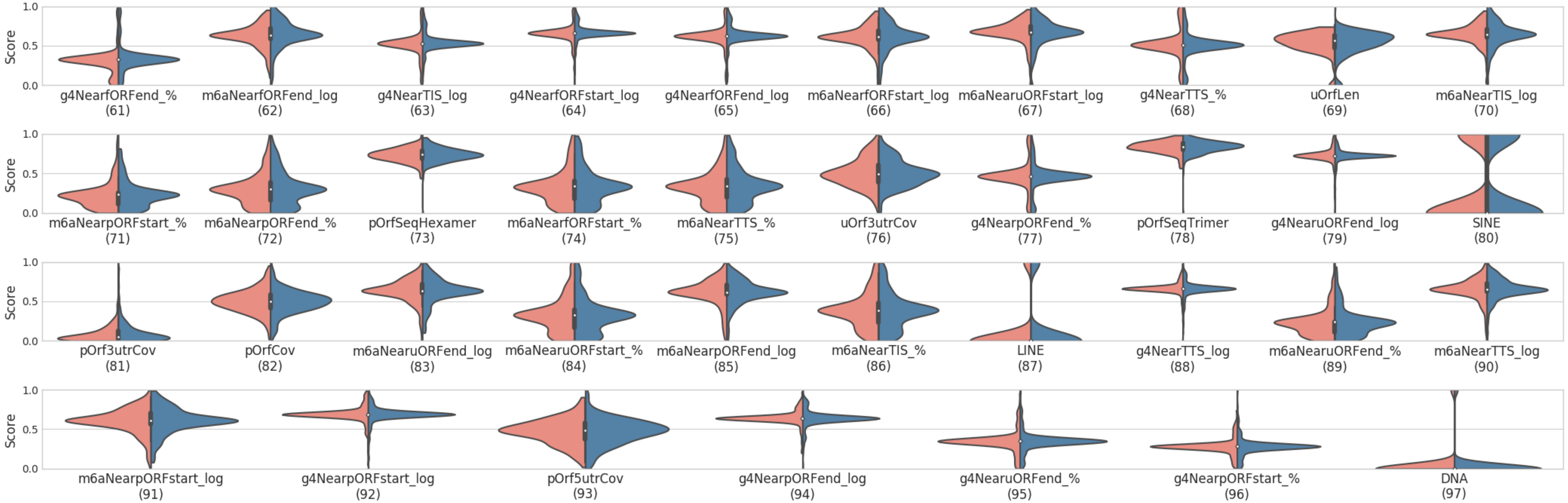

Figure S3 Distribution of all feature scores in mouse (continued).

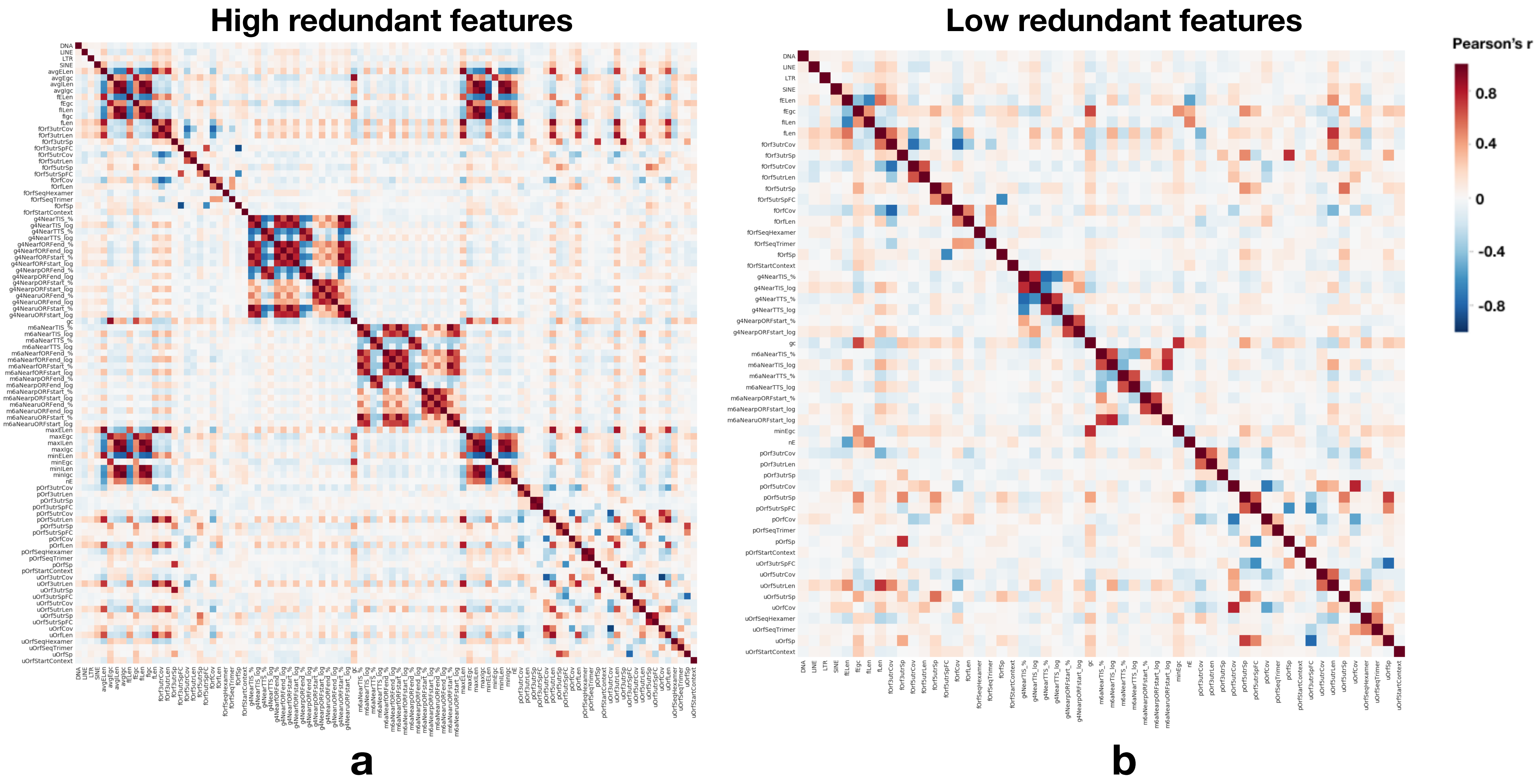

Figure S4 Correlations (r) of features indicates redundant features in mouse. **a** Correlations of all extracted features shows that features of several sub-regions are highly correlated (redundant). **b** After removing high redundant (| r | > 0.8) features, we obtained a low redundant feature set for further analysis in this study.

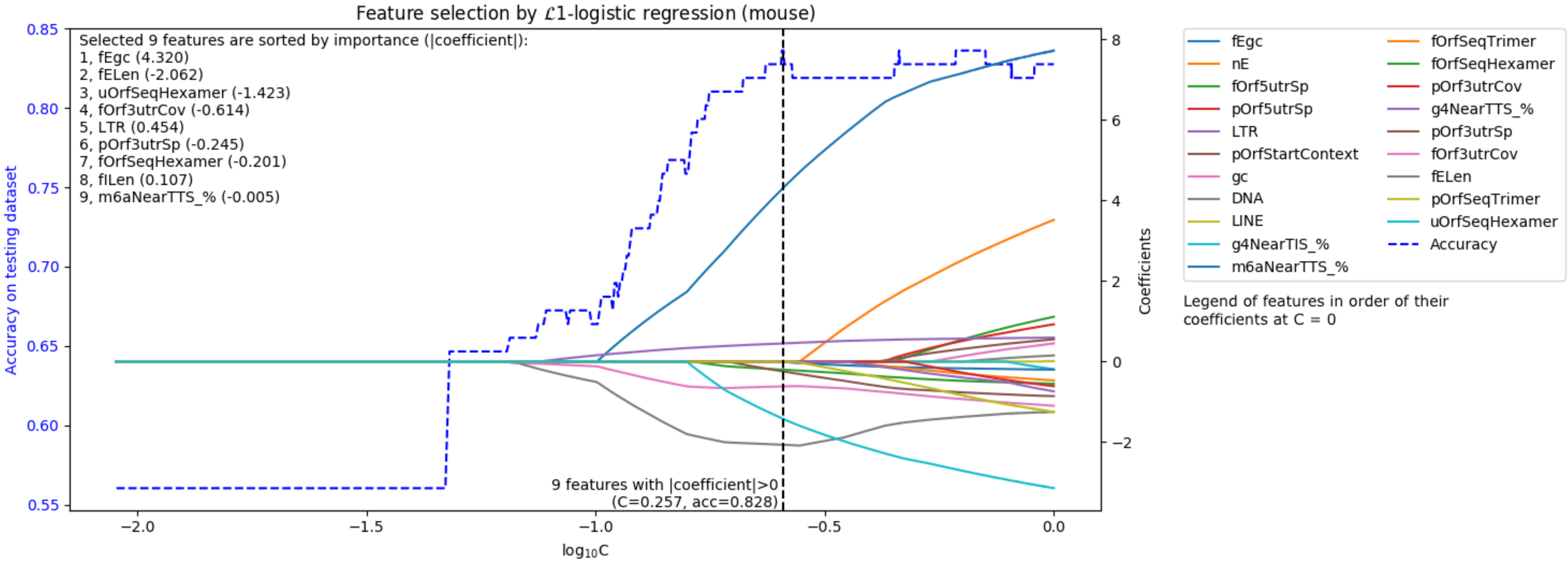

Figure S5 Feature selection by using $\mathcal{L}$1-logistic regression in mouse. Total data was separated into 80% for training the model and 20% for the calculation of accuracy (blue dashed line, left y-axis). On the x-axis, C indicates the inverse of regularization strength. As C is increased, the number of features with non-zero coefficients (right y-axis) is increased and the model becomes more complicated. The black dashed line shows the final model chosen in this study, and outputs 9 features with non-zero coefficients. These features were ranked by the absolute value of coefficient, which represents the importance for prediction, and shown in the upper left.

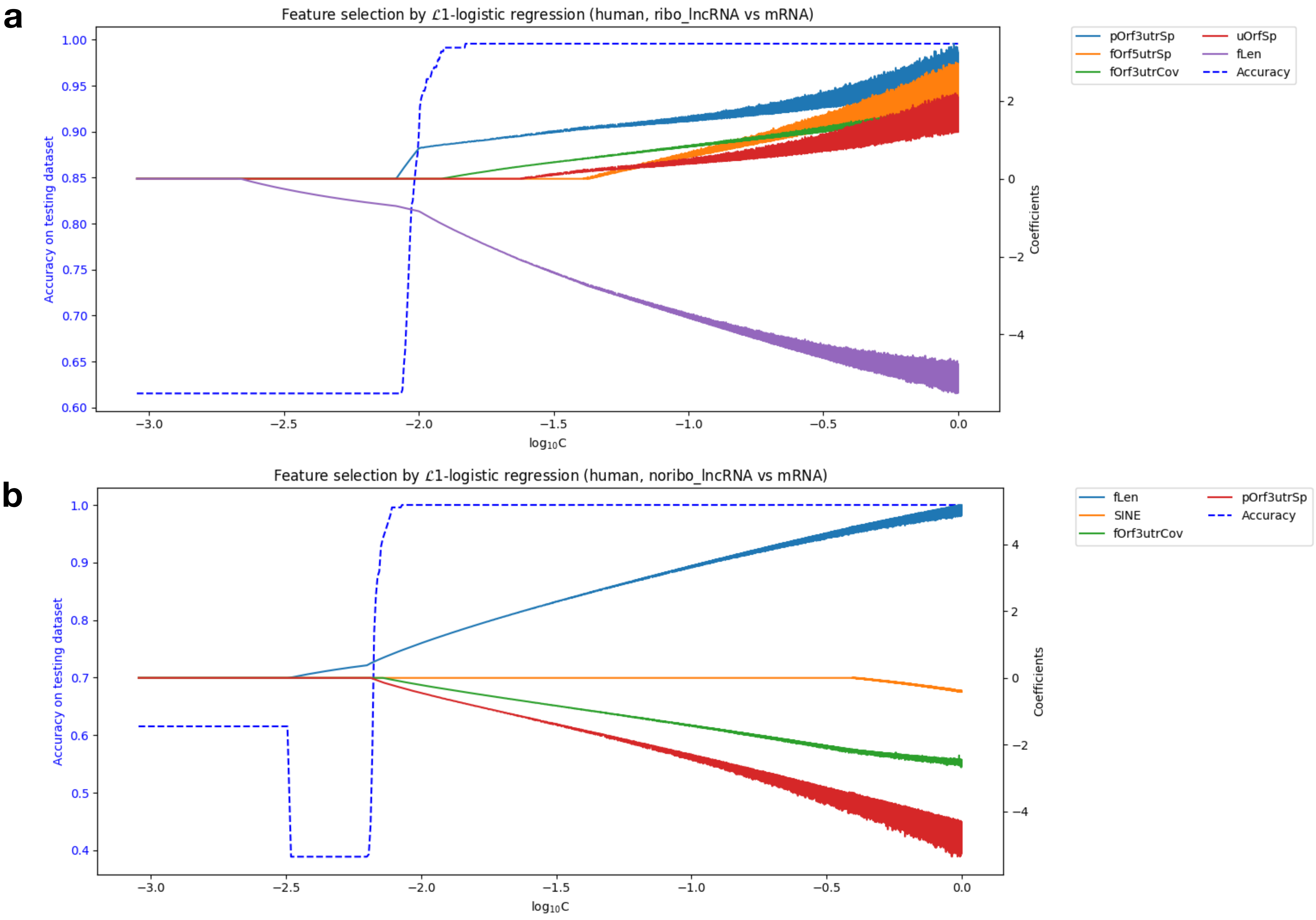

Figure S6 Training $\mathcal{L}1$-logistic regression model on the dataset of **a** ribo-lncRNAs and mRNAs; **b** noribo-lncRNAs and mRNAs in human.

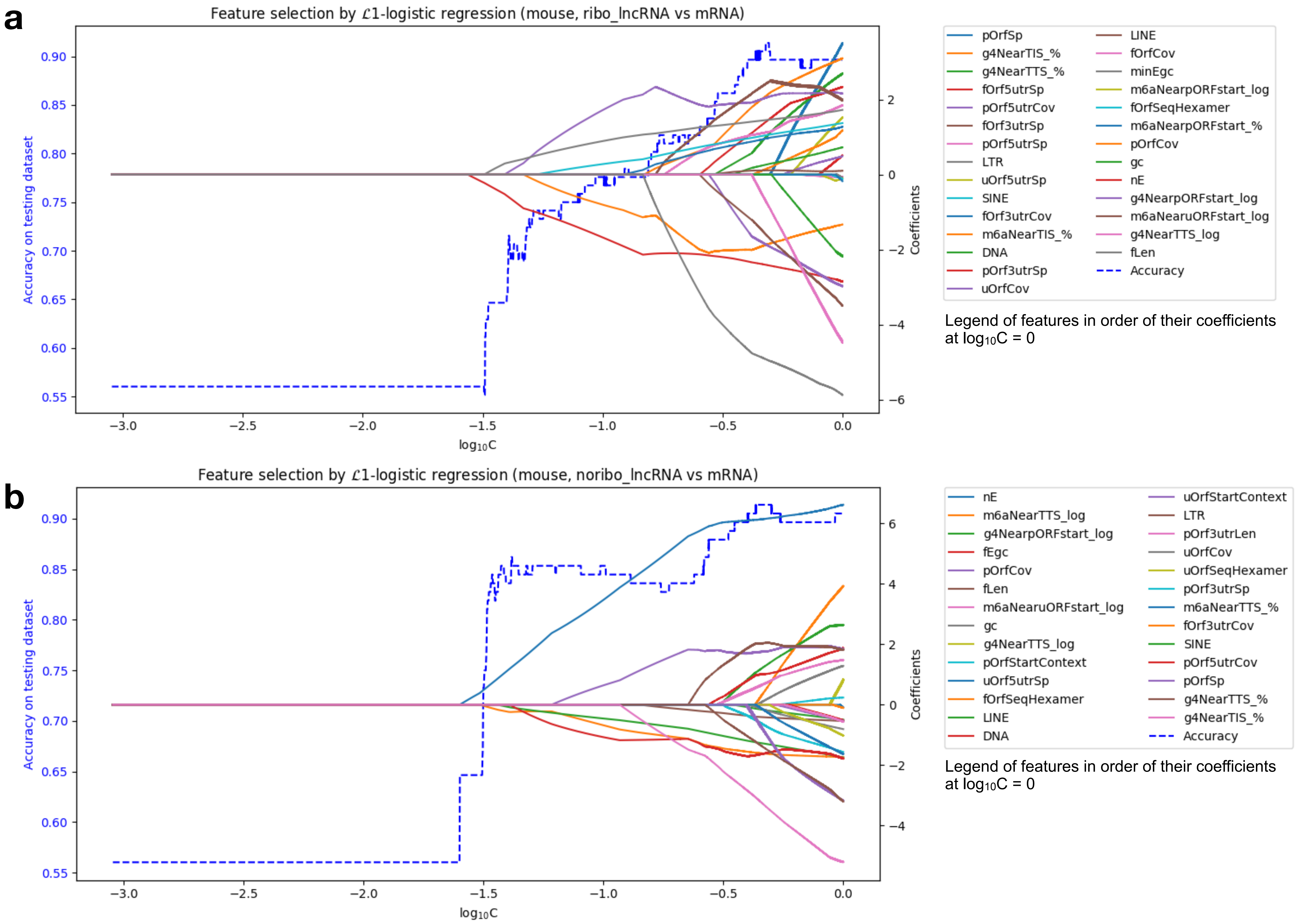

Figure S7 Training $\mathcal{L}$1-logistic regression model on the dataset of **a** ribo-lncRNAs and mRNAs; **b** noribo-lncRNAs and mRNAs in mouse.

Table S1. Sequence features were considered to influence the ribosomal association.

No.	Feature	Description
**Basic**		
1	fLen	Log_{10}(length+1) of the mature lncRNA
2	gc	G+C content of the mature lncRNA
**RNA splicing**		
3	nE	Number of exons
4	fELen	Log_{10}(length+1) of the first exon
5	minELen	Log_{10}(length+1) of the shortest exon
6	maxELen	Log_{10}(length+1) of the longest exon
7	avgELen	Log_{10}(averaged_length+1) of exons
8	fEgc	G+C content of the first exon
9	minEgc	G+C content of the shortest exon
10	maxEgc	G+C content of the longest exon
11	avgEgc	Averaged G+C content of exons
12	fILen	Log_{10}(length+1) of the first intron
13	minILen	Log_{10}(length+1) of the shortest intron
14	maxILen	Log_{10}(length+1) of the longest intron
15	avgILen	Log_{10}(averaged_length+1) of introns
16	fIgc	G+C content of the first intron
17	minIgc	G+C content of the shortest intron
18	maxIgc	G+C content of the longest intron
19	avgIgc	Averaged G+C content of introns
**Putative ORF (pORF: primary ORF; fORF: first ORF; uORF: upstream ORF)**		
20-22	p/f/uOrfLen	Log_{10}(length + 1) of ORF
23-25	p/f/uOrfCov	Percentage of ORF length compared to that of lncRNA
26-28	p/f/uOrf5utrLen	Log_{10}(length + 1) of the upstream region of ORF (5′ UTR)
29-31	p/f/uOrf5utrCov	Percentage of the 5′ UTR length compared to that of lncRNA
32-34	p/f/uOrf3utrLen	Log_{10}(length + 1) of the downstream region of ORF (3′ UTR)
35-37	p/f/uOrf3utrCov	Percentage of the 3′ UTR length compared to that of lncRNA
**K-mer frequency**		
38-40	p/f/uOrfStartContext	Context sore of ORF start
41-43	p/f/uOrfSeqTrimer	Trimer score of ORF

Table S1. Sequence features (continued).

No.	Feature	Description
44-46	p/f/uOrfSeqHexamer	Hexamer score of ORF
**RNA secondary structure**		
47-49	p/f/uOrfSp	Averaged RNA stem probability of ORF
50-52	p/f/uOrf5utrSp	Averaged RNA stem probability of 5′ UTR
53-55	p/f/uOrf5utrSpFC	Ratio of RNA stem probability of 5′UTR to that of ORF
56-58	p/f/uOrf3utrSp	Averaged RNA stem probability of 3′ UTR
59-61	p/f/uOrf3utrSpFC	Ratio of RNA stem probability of 3′UTR to that of ORF
62	g4NearTIS_log	Log_{10}(minimum distance) from G4 to transcription initiation
63	g4NearTTS_log	Log_{10}(minimum distance) from G4 to transcription termination
64-66	g4Near(p/f/u)ORFstart_log	Log_{10}(minimum distance) from G4 to ORF start
67-69	g4Near(p/f/u)ORFend_log	Log_{10}(minimum distance) from G4 to ORF end
70	g4NearTIS_%	Minimum distance from G4 to TIS divided by length of lncRNA
71	g4NearTTS_%	Minimum distance from G4 to TTS divided by length of lncRNA
72-74	g4Near(p/f/u)ORFstart_%	Minimum distance from G4 to ORF start divided by length of lncRNA
75-77	g4Near(p/f/u)ORFend_%	Minimum distance from G4 to ORF end divided by length of lncRNA
**RNA modification**		
78	m6aNearTIS_log	Log_{10}(minimum distance) from m^6A to transcription initiation
79	m6aNearTTS_log	Log_{10}(minimum distance) from m^6A to transcription termination
80-82	m6aNear(p/f/u)ORFstart_log	Log_{10}(minimum distance) from m^6A to ORF start
83-85	m6aNear(p/f/u)ORFend_log	Log_{10}(minimum distance) from m^6A to ORF end
86	m6aNearTIS_%	Minimum distance from m^6A to TIS divided by length of lncRNA
87	m6aNearTTS_%	Minimum distance from m^6A to TTS divided by length of lncRNA
88-90	m6aNear(p/f/u)ORFstart_%	Minimum distance from m^6A to ORF start divided by length of lncRNA
91-93	m6aNear(p/f/u)ORFend_%	Minimum distance from m^6A to ORF end divided by length of lncRNA
**Repeat element**		
94	DNA	Containing DNA transposon or not
95	LINE	Containing LINE element or not
96	LTR	Containing LTR element or not
97	SINE	Containing SINE element or not
98	Retroposon	Containing Retroposon element or not
99	Satellite	Containing Satellite element or not

Table S2. Low-redundant features in human and mouse.

No.	Human	Mouse	No.	Human	Mouse
1	fLen	fLen	31	DNA	DNA
2	gc	gc	32	LINE	LINE
3	nE	nE	33	LTR	LTR
4	fELen	fELen	34	Retroposon	SINE
5	fEgc	fEgc	35	SINE	m6aNearTIS_log
6	fILen	minEgc	36	Satellite	m6aNearTTS_log
7	pOrfCov	fILen	37	m6aNearTIS_log	m6aNearpORFstart_log
8	pOrfSp	pOrfCov	38	m6aNearTTS_log	m6aNearuORFstart_log
9	pOrf5utrCov	pOrfSp	39	m6aNearpORFstart_log	g4NearTIS_log
10	pOrf5utrSp	pOrf5utrCov	40	m6aNearuORFstart_log	g4NearTTS_log
11	pOrf5utrSpFC	pOrf5utrSp	41	g4NearTIS_log	g4NearpORFstart_log
12	pOrf3utrLen	pOrf5utrSpFC	42	g4NearTTS_log	m6aNearTIS_%
13	pOrf3utrCov	pOrf3utrLen	43	g4NearpORFstart_log	m6aNearTTS_%
14	pOrf3utrSp	pOrf3utrCov	44	g4NearfORFend_log	m6aNearpORFstart_%
15	fOrfLen	pOrf3utrSp	45	m6aNearTIS_%	g4NearTIS_%
16	fOrfCov	fOrfLen	46	m6aNearTTS_%	g4NearTTS_%
17	fOrfSp	fOrfCov	47	m6aNearpORFstart_%	g4NearpORFstart_%
18	fOrf5utrLen	fOrfSp	48	g4NearTIS_%	pOrfStartContext
19	fOrf5utrCov	fOrf5utrLen	49	g4NearTTS_%	fOrfStartContext
20	fOrf5utrSp	fOrf5utrCov	50	g4NearpORFstart_%	uOrfStartContext
21	fOrf5utrSpFC	fOrf5utrSp	51	pOrfStartContext	pOrfSeqTrimer
22	fOrf3utrCov	fOrf5utrSpFC	52	fOrfStartContext	fOrfSeqTrimer
23	fOrf3utrSp	fOrf3utrCov	53	uOrfStartContext	fOrfSeqHexamer
24	uOrfCov	fOrf3utrSp	54	pOrfSeqTrimer	uOrfSeqTrimer
25	uOrfSp	uOrfCov	55	pOrfSeqHexamer	uOrfSeqHexamer
26	uOrf5utrLen	uOrfSp	56	fOrfSeqTrimer	
27	uOrf5utrCov	uOrf5utrLen	57	fOrfSeqHexamer	
28	uOrf5utrSp	uOrf5utrCov	58	uOrfSeqTrimer	
29	uOrf5utrSpFC	uOrf5utrSp	59	uOrfSeqHexamer	
30	uOrf3utrSpFC	uOrf3utrSpFC			
